# MALDI-TOF Protein Profiling Reflects Changes in Type 1 Diabetes Patients Depending on the Increased Amount of Adipose Tissue, Poor Control of Diabetes and the Presence of Chronic Complications

**DOI:** 10.3390/ijerph18052263

**Published:** 2021-02-25

**Authors:** Agnieszka Zawada, Dariusz Naskręt, Eliza Matuszewska, Zenon Kokot, Marian Grzymisławski, Dorota Zozulińska-Ziółkiewicz, Agnieszka Dobrowolska, Jan Matysiak

**Affiliations:** 1 Department of Gastroenterology, Dietetics and Internal Medicine, Poznan University of Medical Sciences, 60-355 Poznań, Poland; mariangrzym@ump.edu.pl (M.G.); agdob@ump.edu.pl (A.D.); 2 Department of Internal Medicine and Diabetology, Poznan University of Medical Sciences, 60-834 Poznań, Poland; dnaskret@poczta.onet.pl (D.N.); zozula@box43.pl (D.Z.-Z.); 3 Department of Inorganic and Analytical Chemistry, Poznan University of Medical Sciences, 60-780 Poznań, Poland; eliza.matuszewska@ump.edu.pl (E.M.); jmatysiak@ump.edu.pl (J.M.); 4 Faculty of Health Sciences, Calisia University Kalisz, 62-800 Kalisz, Poland; zkokot@ump.edu.pl

**Keywords:** type 1 diabetes, proteomic profile, obesity, C4 complement

## Abstract

Introduction: Protein profiling allows the determination of the presence of proteins marking various stages of the disease, and differentiates between people at risk of various diseases. In type 1 diabetes, protein profiling had been previously used to find blood markers other than islet autoantibodies to indicate the pancreatic beta cell destruction process and to reflect the progression of type 1 diabetes mellitus (T1DM). However, T1DM is an auto-immune disease and its clinical presentation changes in time of its duration. The aim of the study: To find differences in protein profiles in patients with type 1 diabetes according to diabetes control (HbA1c > 7%) and with presence of diabetic complications or obesity. It may help to identify subgroups of patients who may need a better clinical supervision and individualized treatment. Material and methods: A group of 103 patients with auto-immunologically confirmed T1DM, and meeting the following inclusion criteria: Caucasian race, duration of diabetes >5 years, were used in the study. Criteria of exclusion: past or present cancer (treated with chemo-/radiotherapy), diseases of the liver (ALT > 3 × ULN) except for people with simple hepatic steatosis, chronic renal disease (eGFR < 30 mL/1.73 m^2^/min), and acute inflammation (CRP > 5 mg/dL). The study group was divided in terms of the presence of chronic complications, obesity, or poor metabolic control (HbA1c > 7%). Protein profiling was completed by using the MALDI-TOF MS (matrix-assisted laser desorption/ionization-time of flight mass spectrometry) analyzer. Results: Differentiating proteins were identified in all of the groups. The groups burdened with complications, obesity, and poor metabolic control were characterized by increased levels of fibrinogen, complement C4 and C3. Conclusion: The groups of type 1 diabetes patients burdened with complications, obesity, and poor metabolic control were characterized by increased levels of fibrinogen, complement C4 and C3. Further detailed studies are necessary to determine more subtle changes in the proteomic profile of patients with type 1 diabetes.

## 1. Introduction

Patients with T1DM constitute about 5–10% of the population of patients with the disease. T1DM incidence is ranging from under one to up to 60 new cases per 100,000 children per year in the under-15 age-group. Incidence varies by region. Peak incidence is usually in the 10–14-year age [1]. T1DM is also commonly recognized in adults; over half of the new cases of T1D were ≥ 20 years of age in Scotland in 2018. However, the condition often affects young people and children who will have to carefully control their glycemia for many years to prevent complications and lead a normal lifestyle. For that reason, appropriate treatment from the moment of the disease’s development and preventing complications involving micro- and macroagniopathies is important. Moreover, type 1 diabetic patients are at risk of becoming overweight and obese. This risk is increased with the duration of diabetes, which is associated with high daily doses of insulin. For that reason, optimum glycaemia values should be strived for, individually adjusted minimal effective doses of insulin should be administered, and most importantly, patients should be properly educated about their condition [2]. The pathophysiology of T1DM is complex. In people with a genetic predisposition, viral infection or other environmental factors may cause activation of the immune process and clinical manifestation of the disease. In the available literature, protein profiling has been applied to type 1 diabetes to find blood markers other than islet autoantibodies to indicate the pancreatic beta cell destruction process and to reflect the progression of T1DM [3]. Other works have been performed using a mass spectrometric analysis to generate a proteomic profile of protein abundance and post-translational modifications in the aorta and kidneys of diabetic rats [4]. The majority of studies using protein profiling have determined differences in the amount of individual proteins between groups of patients with type 1 diabetes and healthy individuals. Other studies have focused on changes in the protein profile that occur during the autoimmune process during the development of type 1 diabetes and the relationship between protein profiling and the development of complications [5]. Our study is the first differentiating the protein profile in a homogeneous group of patients with autoimmune disease like T1DM. This group is well characterized, selected and treated with a unified method—the method of intensive functional insulin therapy. The patients included in the study were characterized by good compliance. The size of the group is significant, and differences in the protein profile determine the detail of the study in such a homogeneous group of patients. In this study, we focused on demonstrating the difference in protein composition in patients with T1DM, depending on the characteristics acquired during the course of the disease, such as excess body weight or the presence of complications. Demonstration of a difference in the protein profile in subgroup of T1DM patients may be clinically relevant.

In our study, we wanted to find out the differences in protein profiles in patients with T1DM related to the presence of diabetic complications, obesity, and diabetes control depending on the value of glycated hemoglobin > 7%. The demonstration of a difference in the protein profile due to presence of complications and obesity in T1DM patients may help to identify subgroups of patients who may need a better clinical supervision and individualized treatment.

## 2. Materials and Methods

### 2.1. Study Groups

The study was carried out on a group of 103 patients with T1DM treated in the Department of Gastroenterology, Dietetics, and Internal Diseases, and the Department of Internal Medicine and Diabetology in 2016–2020, who met the defined inclusion criteria. The inclusion criteria were T1DM confirmed by the presence of ICA, IA2, and GAD auto-antibodies; treated with intensive functional insulin therapy or with multiple injections of constant insulin doses; age > 18 years; Caucasian race; duration of diabetes > five years; and patient informed consent to participation in the study. The exclusion criteria were other types of diabetes; past or present cancer treatment with radio-or chemotherapy; liver diseases (ALT > 3 × ULN), except for people with simple hepatic steatosis; chronic renal disease (eGFR < 30 mL/1.73 m^2^/min); acute inflammation (CRP > 5 mg/dL); status post ischemic or haemorrhagic cerebral stroke (<6 months); unstable ischemic heart disease (status post STEMI with the implantation of drug-eluting stents, non-STEMI < 12 months); and other diseases (mental disorders, nutritional disorders, e.g., anorexia, bulimia), other treatment (antibiotic therapy < 3 months, and current steroid therapy), and dependence on alcohol (consumption of more than one unit of alcohol a day). The study group consisted of people with T1DM mostly treated with intensive functional insulin therapy, with mean age 34 years. The duration of diabetes was significantly longer than the inclusion criterion. All patients had a history of T1DM longer than 12 years. The clinical characteristics of the study population are given in Table 1. 

### 2.2. Methods

The study group was divided into three subgroups according to the presence of:

1. Obesity (BMI < 30 vs. BMI > 30 kg/m^2^): BMI was calculated from the formula of the ratio of body weight to squared height.
2. Presence of diabetic complications (retinopathy, nephropathy, and neuropathy, all complications or any of them)
3. HbA1c value < 7% and > 7%: A reasonable A1C goal according to American Diabetes Association Guidelines 2020 for many nonpregnant adults [4].

#### 2.2.1. Assessment of Diabetic Complications

##### Assessment of Diabetic Kidney Disease (DKD)

The elimination of albumin with urine was assessed based on a 12-h urine collection, with a determination of the albumin/creatinine index in the morning sample of urine. Albumin elimination between 30 and 300 mg a day with urine on two of three urine collection days and the albumin/creatinine index of >30 mg/g in morning urine sample were deemed positive albuminuria. Diabetic kidney disease was diagnosed in case of the presence of pathological albuminuria and at least a 10-year history of diabetes, or co-existence of diabetic retinopathy. DKD was divided into stages based on the estimated glomerular filtration rate: G1 stage (Egfr ≥ 90 mL/min/1.73 m^2^), G2 stage (eGFR 60–89 mL/min/1.73 m^2^), G3a stage (eGFR 45–59 mL/min/1.73 m^2^), G3b stage (eGFR 30–45 mL/min/1.73 m^2^), G4 stage (eGFR 15–29 mL/min/1.73 m^2^), and G5 stage (eGFR <15 mL/min/1.73 m^2^ or treatment with dialysis).

##### Assessment of Diabetic Retinopathy

An ophthalmoscopic examination of eye fundus was carried out following dilation of the pupil. Diabetic retinopathy was diagnosed when at least one micro-aneurysm was found in each eye. Diabetic retinopathy was classified acc. to the Polish Diabetes Association as: no evidence of diabetic retinopathy, mild non-proliferative diabetic retinopathy (NPDR), moderate non-proliferative diabetic retinopathy, severe non-proliferative diabetic retinopathy, and proliferative diabetic retinopathy (PDR) [6].

##### Assessment of Diabetic Neuropathy

Diabetic neuropathy was assessed with a 10 g Semmes–Weinstein monofilament test. The vibration sensation was assessed using a tuning fork (128 MHz), and the temperature sensation was assessed using a roller with a metal and plastic tip (Tiptherm), and testing the tarsal reflex. Peripheral neuropathy was assessed based on the presence of two or more components: presence of neuropathy symptoms, impaired touch and/or vibration sensation, or absence of the tarsal reflex.

#### 2.2.2. Laboratory Analysis

Laboratory investigations were done in the “H. Święcicki” Teaching Hospital in Poznan. Laboratory measurement methods: Blood for the tests was drawn from fasting patients, using a puncture of the vein in the elbow pit. Biological materials used for the tests were full blood, plasma, and serum.

Glycated hemoglobin level (HbA1c) was assessed using the HPLC method (normal range: 4.8–7%).

Lipid profile: parameters of lipid metabolism (total cholesterol—TCh, HDL-ch fraction, LDL-ch fraction, and TAG in serum) were assayed using the standard method (laboratory standards: TCh: 130–200 mg/dL; 3.3–5.2 mmol/L, HDL-ch: M: 35–70 mg/dL; 0.9–1.8 mmol/L, HDL-ch: F: 45–80 mg/dL; 1.1–2.0 mmol/L, LDL-ch: 60–130 mg/dL; 1.5–3.4 mmol/L, TAG: 30–150 mg/dL; 0.3–1.7 mmol/L). Non-HDL-ch cholesterol level was also calculated using the formula: non-HDL-ch = total cholesterol—HDL-ch. The TAG/HDL-ch ratio was also calculated. Renal parameters: serum creatinine, normal range: F < 0.9 mg/dL, M < 1.2 mg/dL, and the estimated glomerular filtration rate (eGFR) was calculated acc. to the Modification of the Diet Renal Disease Study Equation (MDRD), normal range: 90–120 mL/min/1.73 m². Liver parameters: serum AST activity (reference values: F: 10–31 U/L, M: 10–35 U/L) and serum ALT values (reference values: F: 10–34 U/L, M:10–45 U/L) using the standard method.

#### 2.2.3. MALDI-TOF MS Profiling

Protein profiling was performed in order to find proteins that were characteristic for people with diabetes complicated by obesity and inferior metabolic control (HbA1c > 7%) MALDI-TOF MS profiling and identification of discriminatory proteins and peptides.

##### Sample Pretreatment

Serum samples obtained from patients diagnosed with diabetes were purified and concentrated before mass spectrometry analyses. Pretreatment of the biological material was performed with ZipTip C18 (Millipore, Bedford, MA, USA) reverse phase chromatography micropipette tips. Then, 2 µL of each serum sample was mixed with 8 µL of 0.1% trifluoroacetic acid (TFA) in water. Mixtures were then loaded onto ZipTip tips according to the manufacturer’s protocol. For the tips conditioning, acetonitrile (ACN) and 0.1% TFA were used. Adsorbed peptides were first washed with 0.1% TFA in water and then eluted with 50% ACN in 0.1% TFA.

##### MALDI-TOF MS Analysis

For the MALDI-TOF MS (matrix-assisted laser desorption/ionization-time of flight mass spectrometry) analysis, 1 µL of each eluent obtained from the ZipTip sample pretreatment was mixed with 10 µL of matrix solution (0.3 g/L α-cyano-4-hydroxycinnamic acid (HCCA) in a 2:1 mixture of ethanol/acetone, *v/v*). The mixtures were then manually spotted onto the Anchor Chip Standard (Bruker Daltonics, Bremen, Germany) target plate in triplicates. MS analysis was performed in a linear-positive mode with Ultrafle Xtreme (Bruker Daltonics, Bremen, Germany) mass spectrometer. Ions were analyzed in the range of m/z 1000–10,000. Every MS spectrum was acquired from an average 2000 laser shots per sample. External calibration was performed with a mixture of Protein Calibration Standard I and Peptide Calibration Standard (Bruker Daltonics, Bremen, Germany) in 5:1 (*v/v*) ratio. The average mass deviation from the reference masses did not exceed 100 ppm. MALDI-TOF MS analysis was performed with the following parameters: ion source 1, 25.09 kV; ion source 2, 23.80 kV; pulsed ion extraction, 260 ns, lens 6.40 kV, matrix suppression cut off m/z 700. FlexControl 3.4 (Bruker Daltonics, Bremen, Germany) software was applied for collection and processing of the MS spectra. For the analysis of recorded MS data, Clin Pro Tools 3.0 (Bruker Daltonics, Bremen, Germany) software was applied. Statistical analyses were performed with classification algorithms (quick classifier (QC), genetic algorithm (GA), and supervised neural network (SNN)) and ROC curves [7]. QC is a chemometric algorithm that calculates peaks’ average areas and uses *p*-values for peaks classification. GA, based on the process of natural selection and the idea of the evolution of the fittest individual, allows determination of the discriminatory peaks. SNN-based algorithm chooses MS data with features characteristic to studied groups and according to them, classifies spectra to the proper group. Parameters of cross-validation, external validation, and recognition capability were calculated for each algorithm. The statistical analysis resulted in the depiction of peptide candidates for the subsequent identification.

##### nanoLC MALDI-TOF/TOF MS Identification of Discriminatory Peaks

Identification of the peaks discriminating between the studied groups was performed with nanoLC-MALDI-TOF/TOF MS (nano-liquid chromatography-matrix-assisted laser desorption/ionization–time of flight/time of flight mass spectrometry) system. The samples were pretreated with ZipTip pipette tips and subjected to nanoLC separation. The nanoLC set consisted of EASY-nLC II (Bruker Daltonics, Bremen, Germany) nanoflow HPLC system and Proteineer-fc II (Bruker Daltonics, Bremen, Germany) collector of fractions. The nanoLC system parts were NS-MP-10 BioSphere C18 (NanoSeparations, Nieuwkoop, The Netherlands) trap column (20 mm × 100 µm I.D., particle size 5 µm, pore size 120 Å) and an Acclaim PepMap 100 (Thermo Scientific, Sunnyvale, CA, USA) column (150 mm × 75 µm I.D., particle size 3 µm, pore size 100 Å). The gradient elution method was set on 2–50% of ACN in 96 min (mobile phase A—0.05% TFA in water, mobile phase B—0.05% TFA in 90% ACN). The flow rate for separation was 300 nL/min, and the volume of the sample eluent injected into the chromatography column was 4 µL. From nanoLC separation, in total, 384 separated fractions were obtained. Each of them was mixed with a matrix solution (36 µL of HCCA saturated solution in 0.1% TFA and acetonitrile (90:10 *v/v*), 748 µL of acetonitrile and 0.1% TFA (95:5 *v/v*) mixture, 8 µL of 10% TFA, and 8 µL of 100 mM ammonium phosphate) and spotted automatically onto the AnchorChip Standard (Bruker Daltonics, Bremen, Germany) target plate by the collector of fractions. HyStar 3.2 (Bruker Daltonics, Bremen, Germany) software was used for the nanoLC system operating. For the MS analysis, UltrafleXtreme (Bruker Daltonics, Bremen, Germany) mass spectrometer working in a reflector mode in the range of m/z 700–3500 was used. External calibration was performed with a mixture of Peptide Calibration Standard (Bruker Daltonics, Bremen, Germany). A list of the precursor ions for the identification was established with WARP-LC (Bruker Daltonics, Bremen, Germany) software. Applied settings for MS and MS/MS mode were ion source 1, 7.50 kV; ion source 2, 6.75 kV; reflectron 1, 29.50 kV; reflectron 2, 14.00 kV; lens, 3.50 kV; lift 1, 19.00 kV; lift 2, 3.00 kV; and pulsed ion extraction time, 80 ns. For the spectra acquisition, processing and evaluation FlexControl 3.4, FlexAnalysis 3.4, and BioTools 3.2 (Bruker Daltonics, Bremen, Germany) software were used. For the identification of discriminative proteins and peptides, a SwissProt database and Mascot 2.4.1 search engine with taxonomic restriction to *Homo sapiens* were applied. The protein search parameters were as follows: fragment ion mass tolerance m/z ± 0.7, precursor ion mass tolerance ± 50 ppm, peptide charge + 1, and monoisotopic mass.

#### 2.2.4. Statistical Analysis

The statistical analysis was performed using Statistica PL version 13.3. The conformity of the interval data distribution with the normal distribution was assessed using the Kolmogorov–Smirnov test. In most of the data, no normal distribution was observed. In the analysis, a statistical method for non-parametric variables, Mann–Whitney’s U-test, was used. The results were presented as numbers and percentages, as well as medians and interquartile range (IQR). The value of *p* < 0.05 was assumed to be statistically significant. 

## 3. Results

Incidence of complications are presented in Table 2. The presence of one complication qualified the patient to a group with complications.

Characteristics of the study group according to presence of excess body fat, complication, and value of HbA1c are presented in Table 3.

**Figure 1:**
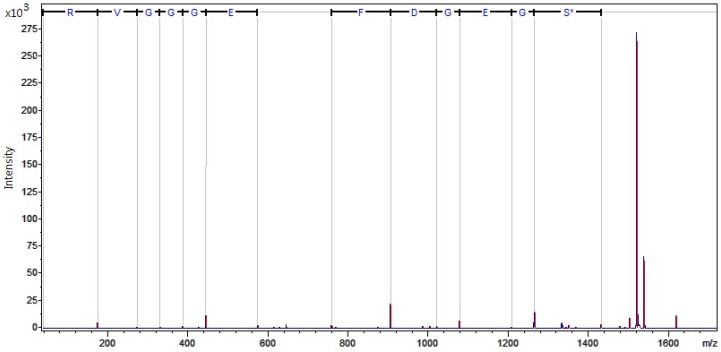
Fragmentation spectrum of peak 1617.79, identified as fibrinogen alpha chain.

**Figure 2:**
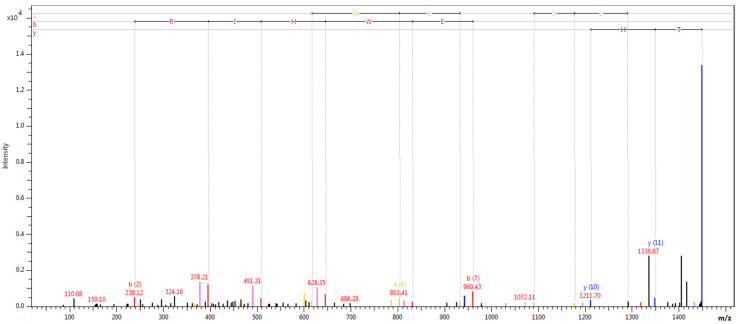
Fragmentation spectrum of peak 1449.61, identified as complement C4A.

**Figure 3:**
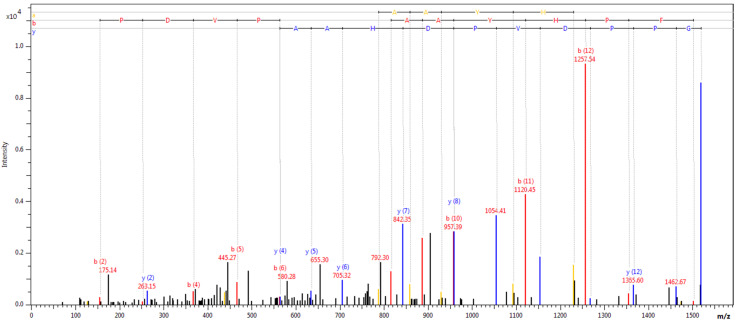
Fragmentation spectrum of peak 1519.99, identified as complement C3.

The results of MALDI-TOF MS analysis are presented in Table 4, Table 5, and Table 6 according to division due to excess fat, diabetes control (HbA1c > 7%), and presence of diabetic complication. Spectra recorded for fibrinogen alpha chain, complement C3, and complement C4A are presented in Figure 1, Figure 2, and Figure 3.

## 4. Discussion

There are not many studies that would analyze the protein profile in patients with T1DM. In our study, we wanted to find differences in metabolic profile in a homogeneous group of people with T1DM in accordance with the presence of diabetic complications, obesity, and diabetes control.

It is well known that the metabolic control of diabetes (mean glucose levels, glycated haemoglobin value, and the time when glucose levels does not remain within their normal ranges) is directly associated with the risk of the development of chronic complications [8,9]. However, there are diabetic patients who do not rapidly develop any chronic complications despite having an inferior diabetes control [10]. It is essential to find proteomic features that could allow for the differentiation of patients with the good and poor control of diabetes, which could increase the risk and accelerate the development of chronic complications. Increasing the level of C3, C4, and fibrinogen in the blood may lead the diabetologist to a more frequent diabetic follow-up visit, patient’s compliance, and better glycemic control. This may inhibit the progression of chronic complications.

Based on the available literature, we were the first to assess the protein/peptide profiles in a group of patients with T1DM, depending on the presence of complications: obesity and metabolic imbalance. Various proteins were identified in all groups of patients, but changed levels of fibrinogen complement C4 and C3 were a common element found in groups burdened with obesity and poor metabolic control. Ostergaard et al. observed that circulating a complement activation product C3a level was increased in streptozotocin-induced diabetic mice as compared to control mice [11]. However, the authors did not perform protein profiling in relation to HbA1c value or the presence of complications of diabetes. It is also stressed that the complement system was emerging as a new potential target in diabetic kidney disease. In our study, we also found a changed level of C3a complement in all groups of patients with excess fat (EBF excess body fat ), complication (presence of complication), and a higher value of HbA1c (HbA1c >7%). The presence of these proteomic markers could be connected with inappropriate diabetes treatment.

Despite the fact that occurrence of T1DM is associated with a genetic predisposition, its development is influenced by various environmental factors; the exact etiology of the disease is not fully known. HLA antigens are proteins found on the body’s cells’ surface and constitute a specific system for every human being. In white people, the presence of the HLA-DR3 and HLA-DR4 genotypes predisposes to the development of diabetes. However, even in people with this genetic predisposition, it is uncertain whether they will develop T1DM. This is dependent on the presence of factors initiating the trigger of an autoimmune process (e.g., severe infection, which initiates the process of destruction of the pancreatic beta cells). This is confirmed by studies carried out in identical twins. With the presence of T1DM in one of the twins, the probability of developing the other is 50 percent. The presence of genetic determinants is not a standard area of study in patients with T1DM, but it can affect changes in the protein profile between individual patients.

The presence of antibodies is associated with the development of type 1 diabetes mellitus, but research programs, including DASP—Diabetes Antibody Standardization Program and Environmental Determinants of Diabetes in Young Consortium, confirm that not all islet auto-antibody-positive subjects progress to T1DM.

Burch et al. attempted to identify new protein markers present in the pancreas of T1DM and type 2 diabetes (T2DM) patients, and in diabetes-free individuals who are carriers of antibodies characteristic for the disease [12]. The study identified proteins allowing differentiation between type 1 and type 2 diabetes in pancreatic tissue. Additionally, proteins’ characteristics for the immunological process, independent on hyperglycemia, were identified by their absence in patients with T2DM. Among proteins differentiating between patients with T1DM and non-diabetic patients, there are complement C5, C7, C8, and C9. On the other hand, complement C3 and C9 proteins ensure differentiation between non-diabetic individuals with positive antibodies and non-diabetic individuals without antibodies [12]. In our study, complement C3, C4, and fibrinogen were proteins that differentiated the group of T1DM patients into subgroups with good (HbA1c < 7%) and poor (HbA1c > 7%) metabolic control and the occurrence of complications.

In a similar study, Nyalwidhe et al. indicated five proteins, including the C9 factor, playing an important role in the development of immunization, inflammation, and metabolic control processes in the course of the development of T1DM [13].

Comparing the metabolomic profile of patients with T1DM to healthy individuals and T2DM patients, Zhang et al. found differences in 33 peptides [14]. Among those proteins, complement C3 showed a significant down-regulation. Notably, peptides from the C1 inhibitor demonstrated a significant up-regulation in T1DM but were down-regulated in T2DM compared to healthy controls. C1 inhibitor is well known to regulate the activation of the C1 complex and may play a crucial role in blood coagulation, fibrinolysis, and suppression of inflammation. In de Oliveira’s study performed on patients with T1DM compared to healthy controls, eight serum proteins were identified as being differentially expressed. C4 complement was down-regulated in diabetic patients [15]. Our study only looked at a group of diabetic patients. We did not compare them with the healthy ones and hence the possibility of different conclusions.

However, Rowe et al. found that complement activation occurred in the pancreas of patients with T1DM and that C4d might be a biomarker for T1DM. In this study, pancreatic C4d antigen expression was more prevalent in patients with T1DM than in diabetes-free subjects (diabetes-free with T1DM-associated islet auto-antibodies, auto-antibodies-negative control subjects, or those with T2DM). C4d density did not differ between diabetes-free subjects [16]. However, these studies mark the proteomic differences between healthy and diabetic subjects or type 1 and type 2 diabetes. Our study focused exclusively on type 1 diabetes.

Christine von Toerne in the study of 45 islet auto-antibody-positive and -negative children from the BABYDIAB/BABYDIET birth cohorts performed a proteomic analysis [17]. Two peptides (from apolipoprotein M and apolipoprotein C-IV) were sufficient to discriminate between auto-antibody-positive and auto-antibody-negative children. Hepatocyte growth factor activator, complement factor H, ceruloplasmin, and age predicted progression time to type 1 diabetes significantly better than age alone.

M. García-Ramírez et al. compared the protein profile of vitreous fluid from diabetic patients with proliferative diabetic retinopathy (PDR) to that from non-diabetic patients with idiopathic macular holes. Eight proteins were highly produced in PDR patients in comparison to non-diabetic subjects: zincα2-glycoprotein (ZAG), apolipoprotein (apo) A1, apoH, fibrinogen A, and the complement factors C3, C4b, C9, and factor B [18].

In our previous study on amino-acid profiling in patients subjected to controlled body weight reduction, it was found that levels of 10 AAs (α-amino-n-butyric acid, alanine, citrulline, glutamine, glycine, hydroxyproline, isoleucine, proline, sarcosine, and threonine) were significantly increased after weight loss compared to their values before the program, while aspartic acid level was decreased [19]. This confirms that changes in protein and amino-acid profile constitute an important element in the pathway of metabolic disorders occurring in the course of the development of chronic complications of diabetes. Changes in the protein profile presented in the study may also be a result of improper glycemic control.

Zhi found differences in the protein profiles of 31 proteins between T1DM patients and healthy individuals [20]. At least 21 of 31 proteins could be functionally relevant to T1DM, as they might be involved in innate immunity, inflammation, and immune response (lymphocyte activation and proliferation and glucose regulation). Complement C3b, complement C4, CRP, and adiponectin are four of those proteins. After a final validation, the differentiating value was attributed to adiponectin and myeloperoxidase. Both proteins are commonly known factors associated with the development of metabolic disorders.

## 5. Conclusions

The molecular mechanism underlying diabetes and its progression is not fully known. Proteomic tools are helping to advance our understanding of the origin, onset, development, treatment, and prevention of diabetes. Moreover, proteomic technologies are becoming more specific and sensitive, and their employment is a significant opportunity to expand our knowledge of T1DM. Protein profiling may also have clinical benefits for a patient with type 1 diabetes. Increased levels of C3, C4, and fibrinogen may draw the attention of a diabetologist for more frequent follow-up at the Diabetes Outpatient Clinic. The patient benefit is that individuals with proteomic markers may increase their interest in glycemic control, which may inhibit the progression of chronic complications. To sum up, advances in proteomic approaches and complete sequences of the human proteome will allow us to unravel changes in the proteomic profile of clinical samples from diabetic patients.

## Figures and Tables

**Table 1:** Clinical characteristic of the study group (*n* = 103). Data are presented as medians with interquartile ranges.

Variable	Median (IQR)
Sex [M/F], *n* (%)	50 (48.5)/ 53 (51.5)
Age [y]	34 (30–42)
DD [y]	17 (12–23)
IFI [y]	12 (8–18)
WHR [*n*]	0.9 (0.8–0.9)
BMI [kg/m^2^]	26 (23–29)
TBF [kg]	19 (14–27)
VF [*n*]	5 (3–8)
DDI [µ/kg/d]	0.5 (0.4–0.6)
HbA1c [%]	8 (7–9)
AST [U/L]	19 (16–24)
ALT [U/L]	19 (14–26)
Creatynine, [μmol/L]	80 (71–88)
eGFR [mL/min/1.73 m^2^]	88 (77–90)
hsCRP [mg/dL]	2 (1–3)
T-ch [mmol/L]	48 (42–54)
TAG [mmol/L]	1 (1–2)
HDL-ch [mmol/L]	2 (1–2)
LDL-ch [mmol/L]	3 (2–3)
non-HDL-c [mmol/L]	3 (3–4)

**Abbreviations:** ACR—albumin/creatinine ratio; ALT—alanine aminotransferase; AST—aspartate aminotransferase; BMI—body mass index; DD—diabetes duration; DDI—daily insulin dose, eGFR; estimated glomerular filtration rate; eGDR, HbA1c—glycated hemoglobin A1c; HDL-C—high density lipoprotein cholesterol; LDL-C—low-density lipoprotein cholesterol; TAG—triglycerides; TBF—total body fat; VF—visceral fat; WHR—waist-to-hip ratio. T-Ch—total cholesterol.

**Table 2:** Incidence of complication in the study group.

Complications	Incidence Frequency in Numbers (*n* = 103)	Incidence Frequency as a Ratio (103 = 100%)
Diabetic retinopathy	39	37.9%
Diabetic renal disease	9	8.7%
Autonomic neuropathy	21	20.4%
Peripheral neuropathy	32	31.1%
The group with any complications	54	52.4

**Table 3:** Clinical characteristics of the study groups (excess body fat vs. normal body fat), (diabetes control, HbA1c < 7) and (presence of complication vs. absence of complication). Data are presented as medians with interquartile ranges.

Value	EBF *N* = 46	NBF *N* = 57	EBF/NBF *P* < 0.05	HbA1c > 7% *N* = 80	HbA1c < 7% *N* = 23	HbA1cp < 0.05	PofC *N* = 55	AofC *N* = 48	PofC/ AofC *P* < 0.05
Age [y]	35.0 (31.0–46.0)	33.0 (29.0–40.0)	0.09	34.0 (25.0–40.0)	35.0 (31.0–42.0)	0.64	38.0 (32.0–47.0)	32.0 (24.0–37.0)	0.00
Sex [M/F]	23/23	27/30	0.82	35/45	15/8	0.07	25/30	25/23	0.51
DD [y]	18.0 (12.0–23.5)	16.0 (11.0–23.0)	0.90	15.0 (11.0–20.0)	19.0 (7.0–21.0)	0.38	20.0 (15.0–29.0)	13.0 (8.0–17.0)	0.00
TBF [kg]	27.6 (23.1–33.2)	14.1 (10.9–18.2)	0.00	21.7 (14.1–29.7)	17.3 (11.8–20.0)	0.07	20.0 (14.1–27.1)	18.2 (13.5–25.0)	0.32
BMI [kg/m^2]^	29.3 (27.8–30.6)	23.7 (21.4–25.7)	0.00	27.1 (24.0–29.8)	24.2 (22.1–28.3)	0.06	26.4 (23.4–30.3)	27.1 (23.0–28.8)	0.66
VF [*n*]	8.0 (6.5–10.0)	4.0 (2.0–5.0)	0.00	5.0 (3.0–8.0)	6.0 (3.0–7.0)	0.93	6.0 (4.0–8.0)	5.0 (2.0–7.0)	0.12
WHR [*n*]	0.9 (0.8–0.9)	0.8 (0.8–0.9)	0.00	0.9 (0.8–0.9)	0.8 (0.9–1.0)	0.04	6.0 (4.0–8.0)	5.0 (2.0–7.0)	0.05
HbA1c [%]	8.4 (7.3–8.9)	7.8 (6.8–8.9)	0.18	8.9 (8.4–9.8)	6.5 (6.2–6.8)	0.00	7.9 (6.9–9.0)	8.4 (7.2–8.9)	0.75
AST [IU/L]	19.0 (15.5–27.0)	19.0 (16.0–22.0)	0.54	18.0 (16.0–27.0)	19.0 (15.0–22.0)	0.66	19.0 (15.0–24.0)	19.0 (16.0–27.0)	0.38
ALT [IU/L]	21.0 (14.0–28.5)	17.0 (14.0–25.0)	0.13	18.0 (13.0–27.0)	21.0 (15.0–25.0)	0.61	18.0 (14.0–24.0)	21.0 (14.0–29.0)	0.25
Creatinine [µmol/L]	70.7 (61.9–88.4)	77.8 (70.7–88.4)	0.19	70.7 (61.9–79.6)	79.6 (70.7–88.4)	0.14	79.6 (70.7–88.4)	79.6 (70.7–88.4)	0.18
GFR [mL/min/1.72 m^2^]	88.2 (82.2–90.0)	86.0 (75.7–90.0)	0.31	90.0 (75.5–90.0)	84.1 (76.5–90.0)	0.38	83.1 (73.4–90.0)	90.0 (84.5–90.0)	0.01
CRP [mg/dL]	2.2 (1.0–4.5)	1.2 (0.6–2.1)	0.00	2.1 (1.0–4.1)	1.0 (0.4–2.0)	0.03	1.4 (0.7–3.1)	1.7 (0.8–3.1)	0.85
TCh [mmol/L]	4.9 (4.5–5.7)	4.6 (3.9–5.1)	0.00	4.8 (4.2–5.3)	4.7 (4.1–5.5)	0.96	4.9 (4.3–5.4)	4.6 (4.0–5.2)	0.31
TAG [mmol/L]	1.2 (1.0–1.8)	0.9 (0.7–1.3)	0.00	1.1 (0.9–1.7)	0.9 (0.7–1.2)	0.00	1.3 (0.9–1.5)	1.3 (0.8–1.4)	0.45
HDL-ch [mmol/L]	1.5 (1.3–1.9)	1.7 (1.4–2.0)	0.11	1.6 (1.3–1.9)	1.7 (1.4–2.3)	0.13	1.6 (1.3–2.0)	1.6 (1.3–2.0)	0.96
LDL-ch [mmol/L]	2.7 (2.5–3.3)	2.4 (1.9–2.9)	0.01	2.6 (2.1–3.1)	2.5 (1.9–3.4)	0.84	2.7 (2.6–3.2)	2.5 (1.9–3.1)	0.18
non-HDL-ch [mmol/L]	3.2 (2.9–4.0)	2.7 (2.2–3.3)	0.00	3.1 (2.6–3.6)	2.7 (2.3–3.9)	0.41	3.2 (2.7–3.6)	2.8 (2.4–3.8)	0.33
ACR [mg/d]	3.5 (2.5–5.2)	3.8 (2.7–5.4)	0.82	3.8 (2.5–5.4)	3.7 (2.9–5.3)	0.88	4.3 (2.9–8.1)	3.3 (2.3–4.3)	0.00

**Abbreviations**: ACR—albumin/creatinine ratio; AofC—absence of complication; ALT—alanine aminotransferase; AST—aspartate aminotransferase; BMI—body mass index; DD—diabetes duration; DDI—daily insulin dose, EBF—excess body fat; eGFR—estimated glomerular filtration rate; eGDR, HbA1c—glycated hemoglobin A1c; HDL-C—high-density lipoprotein cholesterol; LDL-C—low-density lipoprotein cholesterol; NBF—normal body fat; PofC—presence of complication; TAG—triglycerides; TBF—total body fat; VF—visceral fat; WHR—waist-to-hip ratio; T-Ch—total cholesterol.

**Table 4:** Division due to excess body fat.

Division Due to Excess Fat				
Model	Cross Validation [%]	Recognition Capability [%]	External Validation—Correct Classified Part of Valid Spectra [%]—TEST	External Validation—Correct Classified Part of Valid Spectra [%]—CONTROL
**GA**	49.5	93.8	52.9	82.1
**SNN**	59.2	67.1	60.8	38.5
**QC**	58.0	63.4	43.1	74.4
Identified peaks (m/z) classified as discriminatory based on **GA**				
1537.88		fibrinogen alpha chain		
1519.99		complement C3 (oxidation)		
1449.61		complement C4A		
Identified peaks (m/z) classified as discriminatory based on **SNN**				
1519.99		complement C3 (oxidation)		
1537.88		fibrinogen alpha chain		
Identified peaks (m/z) classified as discriminatory based on **QC**				
1435.73		complement C4A		
1449.61		complement C4A		
1519.99		complement C3 (oxidation)		
1537.88		fibrinogen alpha chain		

**Table 5:** Division due to diabetic control (HbA1c > 7%).

Division Due to Diabetes Control (HbA1c > 7%)				
Model	Cross Validation [%]	Recognition Capability [%]	External Validation—Correct Classified Part of Valid Spectra [%]—TEST	External Validation—Correct Classified Part of Valid Spectra [%]—CONTROL
**GA**	62.2	85.8	63.6	30.6
**SNN**	64.6	53.7	0	88.9
**QC**	66.9	66.9	56.8	55.6
Identified peaks (m/z) classified as discriminatory based on **GA**				
1537.88		fibrinogen alpha chain		
1449.61		complement C4A		
1520.00		complement C3 (oxidation)		
Identified peaks (m/z) classified as discriminatory based on **SNN**				
1519.99		complement C3 (oxidation)		
1537.88		fibrinogen alpha chain		
Identified peaks (m/z) classified as discriminatory based on **QC**				
1537.88		fibrinogen alpha chain		

**Table 6:** Division due to diabetes complications.

Division Due to Diabetes Complications				
Model	Cross Validation [%]	Recognition Capability [%]	External Validation—Correct Classified Part of Valid Spectra [%]—TEST	External Validation—Correct Classified Part of Valid Spectra [%]—CONTROL
**GA**	48.2	84.6	46.2	67.6
**SNN**	48.1	65.6	53.8	79.4
**QC**	38.0	63.1	46.2	58.8
Identified peaks (m/z) classified as discriminatory based on **GA**				
1537.88		fibrinogen alpha chain		
1617.79		fibrinogen alpha chain (peak 1537 phosphorylation)		
1435.73		complement C4A		
Identified peaks (m/z) classified as discriminatory based on **SNN**				
1537.88		fibrinogen alpha chain		
1435.73		complement C4A		
1520.00		complement C3 (oxidation)		
1617.79		fibrinogen alpha chain (peak 1537 phosphorylation)		
Identified peaks (m/z) classified as discriminatory based on **QC**				
1537.88		fibrinogen alpha chain		
